# Effective analysis of neurotoxicity and mechanisms of dioctyl terephthalate using network toxicology

**DOI:** 10.3389/fnins.2026.1783807

**Published:** 2026-04-10

**Authors:** Hao Tang, Ting Qin, Zhiwen Yan, Changqing Li, Qinghuan Yang, Yinghong Tang

**Affiliations:** 1 Department of Neurology, The Second Affiliated Hospital of Chongqing Medical University, Chongqing, China; 2 Department of Respiratory Disease, Science City People's Hospital, Chongqing, China; 3 Department of Neurology, The First Affiliated Hospital of Chongqing Medical University, Chongqing, China; 4 Department of Gerontology, The Second Affiliated Hospital of Chongqing Medical University, Chongqing, China

**Keywords:** DOTP, molecular docking, molecular dynamics simulations, network toxicology, neurotoxicity

## Abstract

**Objective:**

Dioctyl terephthalate (DOTP), a widely used plasticizer in food packaging and environmental materials, has raised concerns regarding its potential impact on human health. This study aims to investigate the neurotoxicity-related mechanisms of DOTP through an integrated approach combining network toxicology, molecular dynamics simulations, and *in vivo* validation.

**Methods:**

We retrieved the DOTP chemical structure from PubChem and predicted potential protein targets using the Similarity Ensemble Approach (SEA), SwissTargetPrediction, and SuperPred. We performed protein–protein interaction (PPI) network analysis using STRING and Cytoscape to identify core neurotoxicity-associated targets. Gene Ontology (GO) and Kyoto Encyclopedia of Genes and Genomes (KEGG) pathway enrichment analyses were conducted to characterize the biological relevance of the 90 intersecting targets. We used AutoDock to assess the binding affinity of DOTP to the core proteins, and employed GROMACS for molecular dynamics (MD) simulations to explore the stability and conformational dynamics of the docked complexes. *In vivo* experiments, encompassing behavioral assessments, histological examinations, and molecular assays, were conducted to evaluate the effects of DOTP on neurological function, neuronal integrity, and target pathway dysregulation.

**Results:**

We identified 90 neurotoxicity-related targets, among which EGFR, BCL2, CASP3, MAPK8, TLR4, NFKB1, and MTOR emerged as core nodes within the PPI network. GO and KEGG analyses revealed the involvement of these targets in diverse biological processes, cellular components, molecular functions, and signaling pathways. Molecular docking indicated favorable binding affinities between DOTP and the identified core targets, a finding further supported by MD simulations. Moreover, DOTP-treated mice exhibited significant neurofunctional deficits and neuronal loss, accompanied by profound oxidative stress, neuroinflammation, and apoptotic activation, substantiating its potential neurotoxicity.

**Conclusion:**

Our findings provide a theoretical foundation for understanding the predicted molecular mechanisms of DOTP-induced neurotoxicity. The integration of computational modeling and *in vivo* phenotypic validation suggests that DOTP may pose neurological risks, highlighting the need for further experimental evaluation of plasticizer alternatives.

## Graphical abstract

**Figure:**
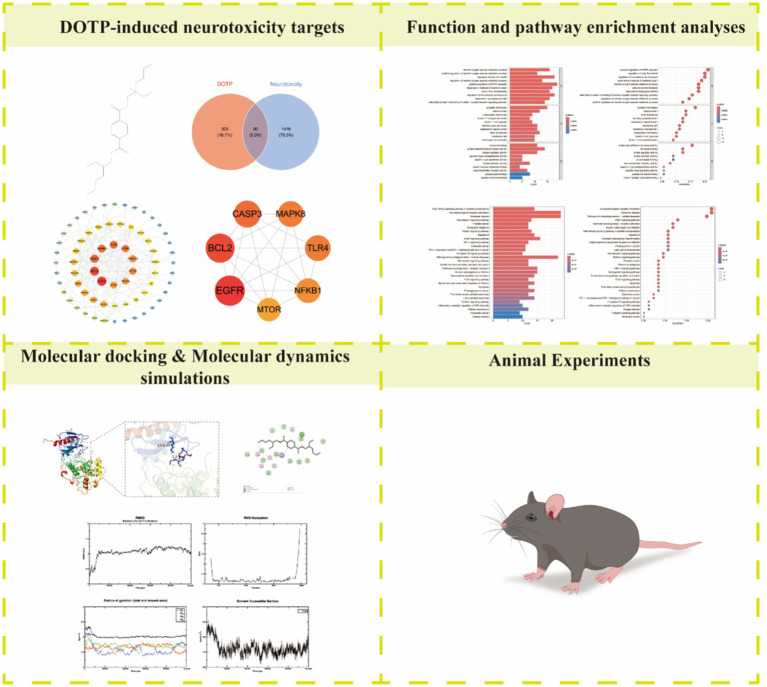
Flowchart of this study. We first identified the potential targets involved in DOTP-induced neurotoxicity and constructed a protein–protein interaction (PPI) network. We then performed functional annotation and pathway enrichment analyses on these targets. Subsequently, we conducted molecular docking and molecular dynamics (MD) simulations to evaluate the interactions between DOTP and key target proteins. Finally, we validated the neurotoxic effects of DOTP *in vivo* using a mouse model.

## Introduction

The rapid phase-out of traditional phthalate plasticizers, such as di(2-ethylhexyl) phthalate (DEHP), driven by their well-documented adverse health effects, has created a pressing demand for safer alternatives in the global plastics industry. Dioctyl terephthalate (DOTP, also known as DEHT or DEHTP) has emerged as a prominent substitute owing to its favorable physicochemical properties, including enhanced weathering resistance, low-temperature flexibility, and an improved environmental profile (Bui et al., 2016; Harmon and Otter, 2022). As regulatory agencies worldwide implement stringent safety assessments for plasticizers, the market penetration of DOTP continues to expand (Silva et al., 2019). Concurrently, evolving regulatory frameworks emphasize the need for comprehensive toxicological evaluations. Consequently, a thorough investigation of the safety profile of DOTP is both timely and imperative to balance industrial innovation with public health (Domínguez-Romero et al., 2023).

Presumed to be biologically benign, DOTP is widely incorporated into consumer and industrial products, including electrical cables, medical devices, and children’s toys. However, the health risks associated with prolonged exposure remain debated (Li et al., 2024). For instance, while perinatal exposure to DOTP (0.75 g/kg) does not notably impair the reproductive system in rats—in contrast to the antiandrogenic effects associated with DEHP and benzyl butyl phthalate (BBP)—studies utilizing fetal testicular cell models reveal that DOTP can reduce testosterone secretion (Faber et al., 2007; Yun and Ji, 2023; Ihn et al., 2024). Furthermore, chronic inhalation of DOTP decreases the spontaneous contractile activity of small intestinal smooth muscle, attenuates adrenergic responsiveness, and enhances sensitivity to cholinergic stimulation (Bobkov et al., 2022). Despite these findings, the potential neurotoxic effects of DOTP remain largely unexplored (Antoniou and Otter, 2024). Given its ubiquity, investigating the impact of DOTP on the nervous system is critical for comprehensively defining its toxicological profile and informing regulatory decisions (Liu et al., 2023).

Elucidating the neurotoxicity of DOTP presents several methodological and conceptual challenges. First, DOTP likely exerts toxic effects through complex, multi-target mechanisms involving numerous proteins and signaling pathways, which traditional single-target assays fail to capture (Valls-Margarit et al., 2023). Second, the structure–activity relationship (SAR) of DOTP remains poorly defined, obscuring how specific molecular features influence its toxicity (Li et al., 2024). Third, existing computational studies often rely on static molecular docking, which cannot account for the dynamic, transient conformational changes governing ligand–protein interactions in biological systems (Salmaso and Moro, 2018).

To address these limitations, we propose an integrative research paradigm combining network toxicology, molecular docking, and molecular dynamics (MD) simulations (Valls-Margarit et al., 2023). The integration of network-based computational models has proven effective in decoding complex toxicological mechanisms, such as the neurotoxicity of agricultural fungicides (Zhu and Lv, 2025b) and the neuropsychiatric adverse effects of clinical medications (Umetsu et al., 2021; Zhu and Lv, 2025a). Building upon these advancements, network toxicology leverages systems biology and bioinformatics to construct interaction maps between DOTP and its potential targets, thereby uncovering multi-target effects and identifying key regulatory nodes. Molecular docking subsequently provides an atomic-level perspective by predicting the binding orientation and affinity of DOTP with neurological proteins, offering preliminary insights into its SAR. Finally, MD simulations capture the dynamic nature of these interactions, revealing binding stability, conformational fluctuations, and interaction kinetics over time (Salmaso and Moro, 2018).

Drawing upon its widespread environmental presence and the established adverse effects of legacy phthalates, we hypothesize that DOTP is not biologically inert, but rather possesses the potential to induce neurotoxicity through complex, multi-target mechanisms (Silva et al., 2019). Therefore, this study aims to comprehensively evaluate the neurotoxic potential of DOTP and decode the underlying molecular pathways. We employed a multiscale paradigm bridging static network toxicology predictions with dynamic MD simulations (100 ns), subsequently anchoring these computational findings with *in vivo* phenotypic validation of behavioral and structural neurotoxicity. To our knowledge, this is the first study to systematically challenge the assumption that DOTP is a neurobiologically safe alternative. By elucidating how DOTP potentially orchestrates oxidative stress, neuroinflammation, and synaptic dysregulation, this study advances the understanding of plasticizer-induced neurotoxicity and provides a theoretical foundation for future environmental risk assessments.

## Materials and methods

### Collection of DOTP targets

We retrieved the standard chemical structure and Simplified Molecular-Input Line-Entry System (SMILES) representation of DOTP from the PubChem database.^1^ Restricting the search to *Homo sapiens*, we identified potential DOTP targets using the Similarity Ensemble Approach (SEA; Keiser et al., 2007),^2^ SuperPred (Gallo et al., 2022),^3^ and SwissTargetPrediction^4^ databases (Daina et al., 2019). After collating the results, we removed duplicate targets to generate a refined list of potential DOTP-associated genes.

### Neurotoxicity-related target network

To identify neurotoxicity-related targets, we queried the GeneCards (Safran et al., 2010),^5^ Therapeutic Target Database (TTD; Zhou et al., 2024),^6^ and Online Mendelian Inheritance in Man (OMIM)^7^ databases (Amberger et al., 2019) using the keyword “neurotoxicity.” To mitigate the inclusion of low-relevance noise while preserving a comprehensive dataset, we selected genes with relevance scores above the median. This dynamic thresholding approach is widely established in network toxicology to balance false positives and false negatives (Wei et al., 2025). It effectively filters out generic background genes while retaining functionally significant targets that might be prematurely excluded by arbitrarily strict absolute cutoffs. We then employed a Venn diagram to identify overlapping targets between DOTP and neurotoxicity, considering these common targets as potential mediators of DOTP-induced neurotoxicity.

### Construction of protein interaction network and screening of core targets

To predict interactions among targets potentially involved in DOTP-induced neurotoxicity, we input the identified overlapping targets into the STRING database. The minimum required interaction score was set to high confidence (> 0.7), and the species was restricted to *Homo sapiens*. The resulting STRING data were imported into Cytoscape (version 3.10.3; Shannon et al., 2003) for visualization and core target screening. Next, we evaluated the topological parameters of the constructed protein–protein interaction (PPI) network using the NetworkAnalyzer tool in Cytoscape. To comprehensively assess the functional importance of each target, we calculated degree centrality (DC), betweenness centrality (BC), and closeness centrality (CC). Finally, we applied the MCODE plugin to identify highly interconnected functional modules within the PPI network (Bader and Hogue, 2003). The highest-scoring cluster was selected for downstream analysis. This criterion was established because the highest-scoring cluster mathematically represents the most robust, densely interconnected, and biologically significant core functional module, thereby effectively filtering out loosely connected peripheral nodes and stochastic network noise.

### Gene function and pathway enrichment analysis of target proteins

To investigate the biological functions of potential targets involved in DOTP-induced neurotoxicity, we performed Gene Ontology (GO) and Kyoto Encyclopedia of Genes and Genomes (KEGG) pathway enrichment analyses using the “clusterProfiler” package (version 4.13.0). The GO analysis assessed biological processes (BP), cellular components (CC), and molecular functions (MF) to reveal key biological activities. In parallel, KEGG enrichment analysis was conducted to identify broad metabolic and signaling pathways. An adjusted *p*-value < 0.05 was applied to ensure statistical reliability. To resolve semantic redundancy among heavily annotated GO terms, we applied the simplify function within the clusterProfiler package with a semantic similarity cutoff of 0.7 (Wu et al., 2021), effectively consolidating highly overlapping child terms. Additionally, to rigorously validate the biological specificity of our findings and establish baseline significance, we conducted a comparative computational study. An identical enrichment pipeline was applied to a randomly generated set of 90 background human genes, serving as a negative control.

### Molecular docking

Molecular docking predicts the preferred orientation of a ligand when binding to a receptor. The 2D structure of DOTP was obtained from the PubChem database and subsequently converted to a 3D conformation using ChemBio3D (version 14.0.0.117), with the output saved in MOL2 format. The 3D structures of the core targets—EGFR (PDB ID: 1 M14), BCL2 (PDB ID: 1G5M), CASP3 (PDB ID: 2DKO), MAPK8 (PDB ID: 4L7F), TLR4 (PDB ID: 2Z62), NFKB1 (PDB ID: 8TQD), and MTOR (PDB ID: 7PED)—were downloaded from the Protein Data Bank.^8^ All selected proteins were originally resolved via X-ray crystallography, ensuring high-resolution structural data. Prior to docking, water molecules and co-crystallized native ligands were removed from the target proteins using PyMOL (version 3.1.3.1). To robustly validate our docking protocol, the extracted native ligands were first re-docked into their respective binding pockets. A root-mean-square deviation (RMSD) of ≤ 2.0 Å between the docked pose and the experimental crystallographic conformation was considered successful validation. Subsequently, AutoDock4 (version 4.2.6) was employed to perform the primary docking calculations. To ensure the reliability of the docking results for the highly hydrophobic DOTP molecule, orthogonal cross-validation was conducted using a second docking engine, AutoDock Vina (version 1.2.7; Eberhardt et al., 2021). Binding energies below 0 kcal/mol indicate spontaneous interactions between the protein and DOTP, while values of ≤ − 5.0 kcal/mol suggest favorable binding and stable occupation of the receptor pocket. Finally, the docking results were visualized using PyMOL and Discovery Studio 2019 to identify the binding sites of DOTP on the target proteins.

### Molecular dynamics simulations

We performed molecular dynamics (MD) simulations using GROMACS (version 2025.0) to investigate the temporal effects of DOTP on the structural stability of its target proteins (Huang et al., 2017). Initial conformations were derived from the optimal binding poses obtained via AutoDock. Proteins were parameterized using the CHARMM36 force field, and ligand topologies were constructed with the GAFF2 force field. Each protein–ligand complex was solvated with the TIP3P water model in a cubic simulation box under periodic boundary conditions. Simulations utilized a leap-frog integrator with a 2-fs time step, running for a total of 50 million steps (100 ns), and trajectory data were recorded every 15,000 steps. The LINCS algorithm constrained all bond lengths. A Verlet cutoff scheme (1.0 nm) was applied for both Coulombic and van der Waals interactions, while long-range electrostatics were handled using the Particle Mesh Ewald (PME) method. System temperature was maintained at 300 K via a V-rescale thermostat (*τ* = 0.1 ps), and pressure was stabilized at 1 bar using a Berendsen barostat with isotropic coupling (τ = 2.0 ps). Three-dimensional periodic boundary conditions and dispersion corrections were applied throughout to ensure accuracy and thermodynamic stability.

### Animal experiment

Male C57BL/6 J mice (8 weeks old, 22–25 g) were sourced from the Animal Experimentation Department of Chongqing Medical University [License No. SCXK (Yu) 2022–0010]. Mice were housed under a 12-h light/dark cycle at 22 ± 2 °C and 50 ± 10% humidity, with ad libitum access to standard chow and water. Following a 7-day acclimation period, animals were randomly assigned to either the control or DOTP group (*n* = 6 per group). Investigators conducting behavioral scoring and histological evaluations were blinded to group allocation.

DOTP (49,234, Sigma-Aldrich, China) was dissolved in purified water to formulate a 200 mg/L stock solution, which was stored at 4 °C (Teng et al., 2024). A working solution (400 μg/L) was freshly prepared every 48 h, protected from light, and provided ad libitum as drinking water for 28 consecutive days; control mice received only purified water. Water bottles were replaced on a fixed schedule to minimize degradation and adsorption, and daily intake was recorded by weighing the bottles to estimate the internal dose (mg/kg/day; Lee et al., 2019; Teng et al., 2024). At the experimental endpoint, mice were euthanized via CO₂ inhalation (30–70% chamber volume/min). Brain tissues were immediately collected, then either fixed for histological assessment or flash-frozen in liquid nitrogen and stored at −80 °C for downstream molecular analyses. All animal procedures were approved by the Ethics Committee of the Second Affiliated Hospital of Chongqing Medical University (Approval No. IACUC-SAHCQMU-2025-0021) and adhered to the ARRIVE guidelines (Percie du Sert et al., 2020).

### Hematoxylin–eosin staining

Following euthanasia, brains were rapidly extracted, fixed in 4% paraformaldehyde for 24 h at room temperature, dehydrated through a graded ethanol series, cleared in xylene, and embedded in paraffin. Coronal sections (5-μm thick) were prepared using a microtome and mounted on charged slides. Sections were then deparaffinized in xylene, rehydrated, and stained using a commercial H&E kit (G1120, Solarbio, China). To ensure consistency across all groups, staining times were strictly standardized: hematoxylin for 5 min, differentiation in 1% acid alcohol for 5–10 s, and bluing in 0.1% ammonia water for 30 s. Finally, sections were counterstained with eosin (1–2 min), dehydrated, cleared, and coverslipped. Images were captured using a bright-field microscope (BX53, Olympus, Japan) at 20 × and 40 × magnification under identical acquisition settings.

### Nissl staining

Paraffin sections were deparaffinized and rehydrated as described above. Nissl staining was conducted using a commercial kit (G1086, Servicebio, China) according to the manufacturer’s protocol. Briefly, sections were incubated in Nissl staining solution for 10 min and differentiated in 95% ethanol for 10–30 s to reduce background noise. Slices were then dehydrated, cleared in xylene, and mounted. Images were acquired using the same microscope and settings applied for H&E staining. All samples were processed in parallel to minimize batch effects.

### Open field test

The open-field test was utilized to evaluate spontaneous locomotor activity. Assessments were conducted in an open-field apparatus (50 cm × 50 cm × 40 cm) featuring a non-reflective white floor and opaque walls. To minimize circadian variations, all behavioral testing occurred during the light phase within a strict time window. Mice were habituated to the testing room for at least 30 min under constant low-noise conditions and 50-lux illumination. Each animal was placed in the center of the arena and allowed to explore freely for 5 min. Locomotor activity was recorded at 25 frames s^−1^ using an automated video tracking system (ANY-maze, Stoelting, United States) with predefined spatial calibration. The arena was thoroughly cleaned with 75% ethanol between trials to eliminate olfactory cues. The primary quantitative endpoints were total distance traveled (m) and mean velocity (m/s), which were calculated automatically using identical detection thresholds for all subjects.

### Biochemical assays and Western blotting

To validate the computational predictions, brain tissues were rapidly excised, washed in pre-chilled phosphate-buffered saline (PBS), minced, and homogenized on ice. For reactive oxygen species (ROS) detection, homogenates were centrifuged at 10,000 × g for 15 min at 4 °C. The resulting supernatants were incubated with a DCFH-DA probe using a commercial ROS Assay Kit (S0033S, Beyotime, China). Fluorescence intensity was subsequently quantified utilizing a microplate reader (excitation: 488 nm; emission: 530 nm). For cytokine profiling, tissues were homogenized in ice-cold saline containing protease inhibitors, subjected to ultrasonic disruption, and centrifuged at 4,000–5,000 × g (4 °C). The concentrations of interleukin-1 beta (IL-1*β*) and tumor necrosis factor-alpha (TNF-*α*) in the supernatants were measured using specific ELISA kits (EK0394, Boster, China; E-EL-M3063, Elabscience, China) following the manufacturers’ instructions. All biochemical measurements were normalized to total protein concentrations, as determined by a bicinchoninic acid (BCA) assay.

For Western blot analysis, total proteins were extracted using RIPA lysis buffer supplemented with protease and phosphatase inhibitors. Equal quantities of protein were resolved via SDS-PAGE and electrotransferred onto polyvinylidene fluoride (PVDF) membranes. After blocking with 5% non-fat milk, the membranes were incubated overnight at 4 °C with primary antibodies against mTOR (T55306S), p-mTOR (T56571S), EGFR (T55112S), p-EGFR (T55232S), and cleaved caspase-3 (TA7022S; all from Abmart, China). Beta-actin (β-actin; 66,009-1-Ig, Proteintech, China) served as the internal loading control. Membranes were then incubated with appropriate HRP-conjugated secondary antibodies. Protein bands were visualized using an enhanced chemiluminescence (ECL) detection system, and relative protein expression levels were quantified using ImageJ software.

### Statistical analysis

Statistical analyses were performed using GraphPad Prism (version 9.5.0, GraphPad Software, United States). Quantitative data are expressed as the mean ± standard error of the mean (SEM) from at least six independent experiments. The normality of data distributions and homogeneity of variance were assessed using the Shapiro–Wilk and F-tests, respectively. For data meeting parametric assumptions, comparisons between the control and DOTP groups were evaluated using a two-tailed unpaired Student’s t-test. Data failing the normality assumption were analyzed using the non-parametric Mann–Whitney U test. A *p*-value < 0.05 was considered statistically significant.

### Quality control

Rigorous quality control measures were implemented across both the computational and *in vivo* modules of this study. For PPI network construction, high-confidence interaction thresholds (> 0.7) were applied against a *Homo sapiens* background. In the MD simulations, adequate system equilibration was confirmed through the stabilization of potential energy and density prior to trajectory analysis. For the animal experiments, strict randomization and blinding protocols governed all behavioral testing and histological evaluations, and tissues were processed in parallel to eliminate batch effects.

## Results

### Identification of DOTP-induced neurotoxicity targets

**Figure 1:**
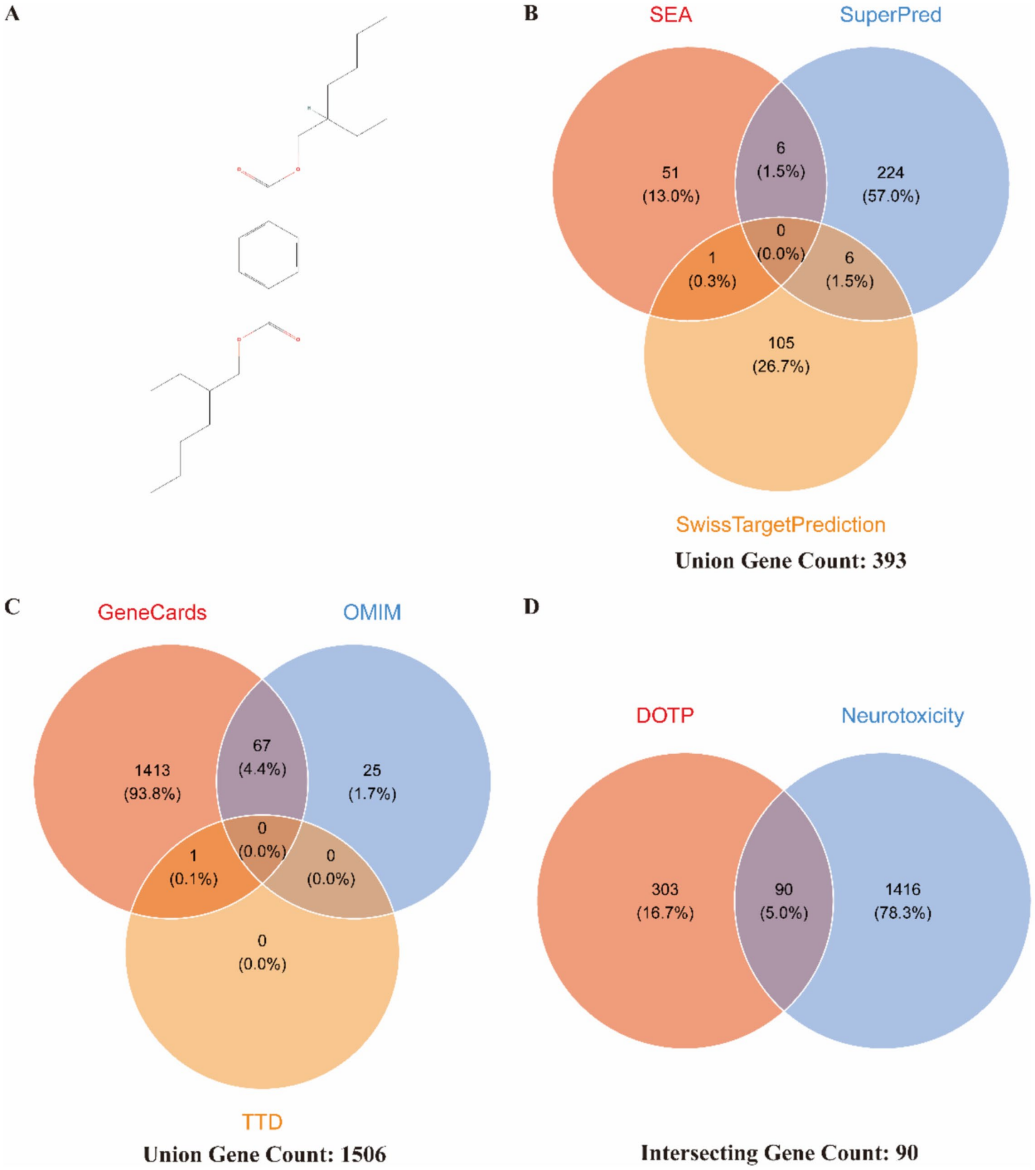
Identification of targets in DOTP-induced neurotoxicity. **(A)** Standard chemical structure of DOTP; **(B)** predicted potential targets of DOTP; **(C)** neurotoxicity-related targets; **(D)** Venn diagram showing the intersection between DOTP targets and neurotoxicity-related targets.

We first obtained the standard chemical structure (Figure 1A) and the SMILES representation [CCCCC(CC)COC(=O)C1 = CC=C(C=C1)C(=O)OCC(CC)CCCC] of DOTP from the PubChem database. A comprehensive search of the SEA, SuperPred, and SwissTargetPrediction databases identified 393 potential DOTP targets (Figure 1B). Additionally, screening the GeneCards, OMIM, and TTD databases yielded 1,506 neurotoxicity-related targets (Figure 1C). After integrating these datasets and removing duplicates, we identified 90 intersecting targets (Supplementary Table S1). A Venn diagram illustrates this intersection (Figure 1D), suggesting that DOTP may induce neurotoxicity via these 90 potential targets.

### Potential targets with the PPI network

**Figure 2:**
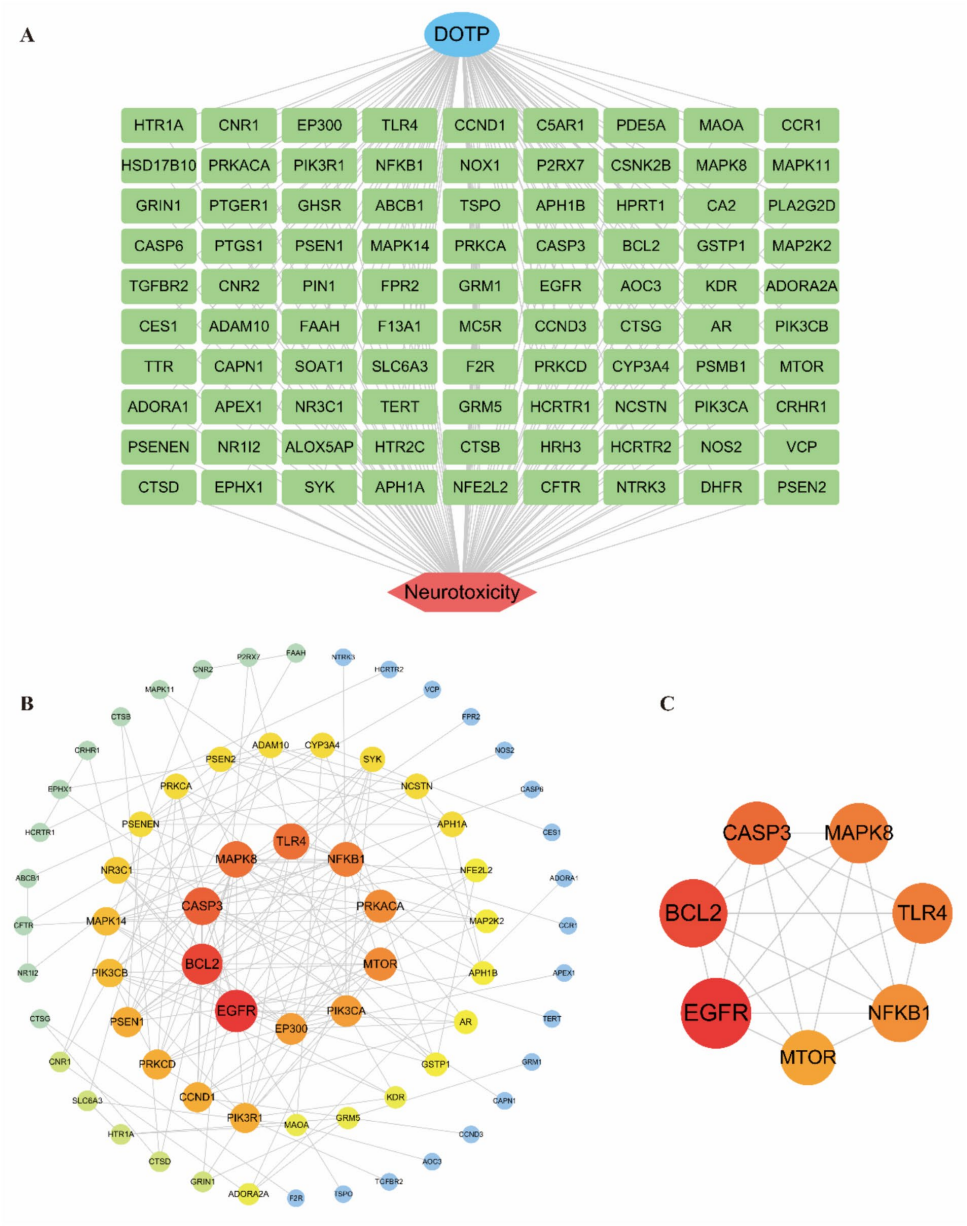
Potential targets with the PPI network. **(A)** Toxin–gene–disease network; **(B)** PPI network of DOTP-induced neurotoxicity targets; **(C)** Core gene clusters identified and visualized by MCODE within the PPI network.

To visualize the potential targets involved in DOTP-induced neurotoxicity, we constructed a toxin–gene–disease network using the 90 intersecting targets (Figure 2A). Next, we built a PPI network using the STRING database, which comprised 90 nodes and 169 edges with an average node degree of 3.76. These data were imported into Cytoscape for visualization and topological analysis (Figure 2B). Node color and size were scaled proportionally to their degree values, indicating that larger, more intensely colored nodes represent higher connectivity. Based on a comprehensive topological assessment integrating DC, BC, and CC (Supplementary Table S2), the top 10 core genes (EGFR, BCL2, CASP3, MAPK8, TLR4, NFKB1, PRKACA, MTOR, PIK3CA, and EP300) emerged as critical hubs and were selected for further analysis. Subsequently, clustering analysis using the MCODE plugin identified the primary functional module. By extracting the highest-scoring cluster, we isolated the most densely interconnected region associated with DOTP-induced neurotoxicity, comprising 7 nodes and 19 edges (Figure 2C). The seed node of this cluster was MAPK8 (Supplementary Table S3), with an MCODE score of 6.333. Analyzing the connectivity of this network provided insights into the molecular mechanisms underlying DOTP-induced neurotoxicity.

### Target function and pathway enrichment analyses

**Figure 3:**
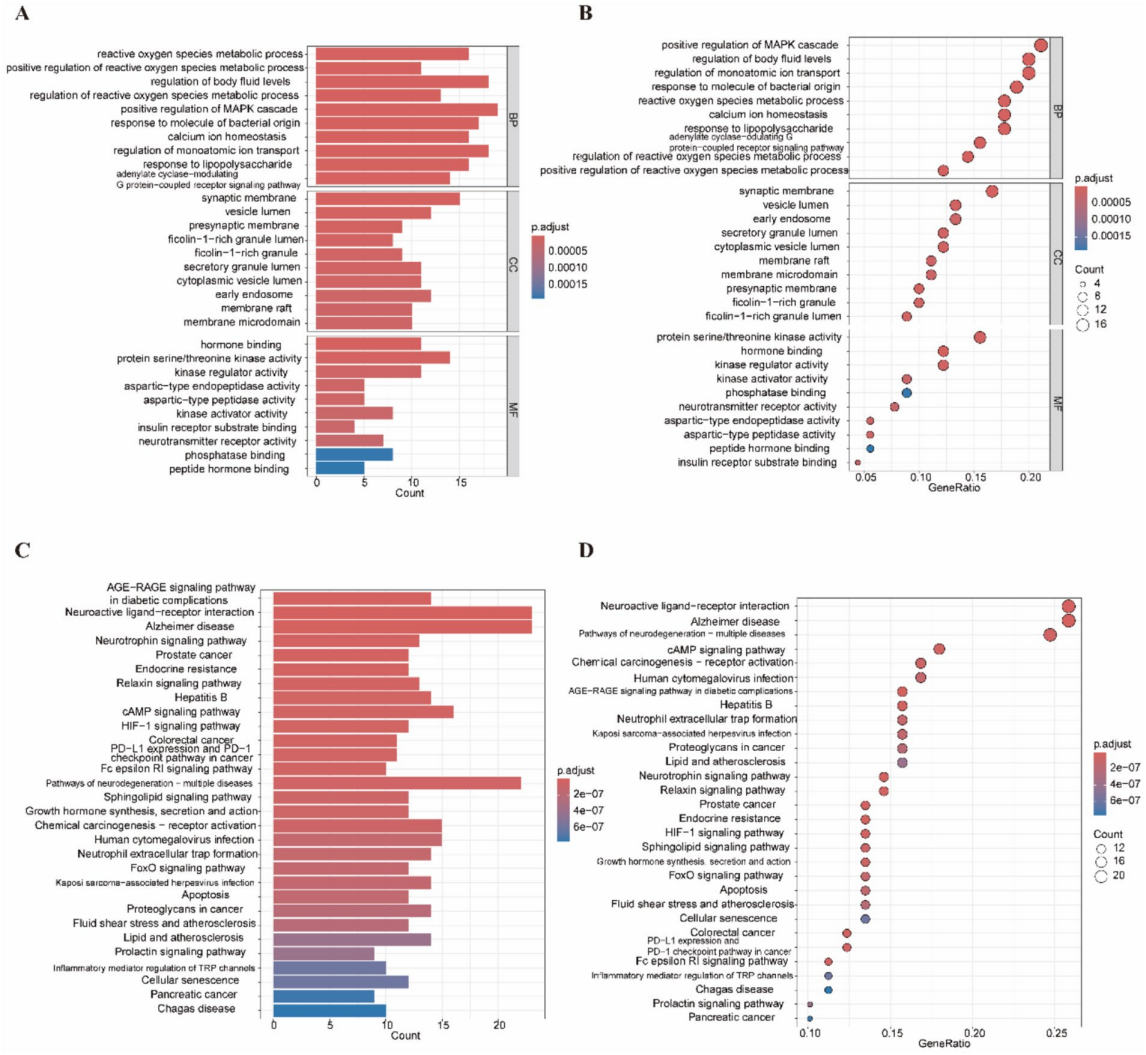
GO and KEGG pathway enrichment analysis of potential targets. **(A)** Histogram of GO enrichment showing the top 10 terms in each category (BP, CC, MF); **(B)** Bubble chart of the top 10 GO terms in each category; bubble size is proportional to gene count and color denotes adjusted *p*-value; **(C)** Bar chart of the top 30 KEGG pathways; bar length represents the number of associated genes; **(D)** Bubble chart of the top 30 significantly enriched KEGG pathways; the *x*-axis shows gene ratio, the *y*-axis lists pathways, bubble size indicates gene count, and color reflects adjusted *p*-value.

To elucidate the mechanisms underlying DOTP-induced neurotoxicity, we performed GO and KEGG enrichment analyses on the 90 intersecting targets. The initial analysis identified 1,345 statistically significant GO terms (Supplementary Table S4). After removing semantically redundant terms to yield distinct functional categories, the refined GO terms were ranked by their adjusted *p*-values, and the top 10 terms for BP, CC, and MF were visualized. A bar chart illustrates the discrete count of genes associated with each term (Figure 3A), while a bubble chart provides a multi-dimensional view integrating gene count, gene ratio, and statistical significance (Figure 3B). Within the BP category, the potential targets were significantly enriched in pathways related to intracellular and extracellular transport and ROS metabolism, suggesting that DOTP may primarily disrupt cellular oxidative defense mechanisms. CC analysis revealed that these genes predominantly operate within membranous structures, including the synaptic membrane. Furthermore, the enriched MFs were closely associated with various protein kinase activities.

KEGG analysis identified 151 significantly enriched signaling pathways (Supplementary Table S5). To display the interrelationships among these pathways, we generated a bar chart showing the 30 most significant KEGG pathways based on gene counts (Figure 3C). This was complemented by a bubble chart illustrating their adjusted p-values and enrichment ratios (Figure 3D). Notably, these targets were highly enriched in neuroactive ligand–receptor interactions, Alzheimer’s disease-related pathways, and neurotrophin signaling.

Crucially, comparative enrichment analysis using a randomly selected set of 90 background genes yielded no significant enrichment in any of these neurological, apoptotic, or stress-related signaling pathways (Supplementary Table S6). This negative computational control confirms that the pathway enrichment of our predicted DOTP targets is specific and not an artifact of generalized network background noise. Overall, these findings indicate that DOTP may promote neurotoxicity by disrupting key cellular processes and signaling pathways crucial for neuronal function and survival.

### Molecular docking of DOTP to the neurotoxicity core targets

**Figure 4:**
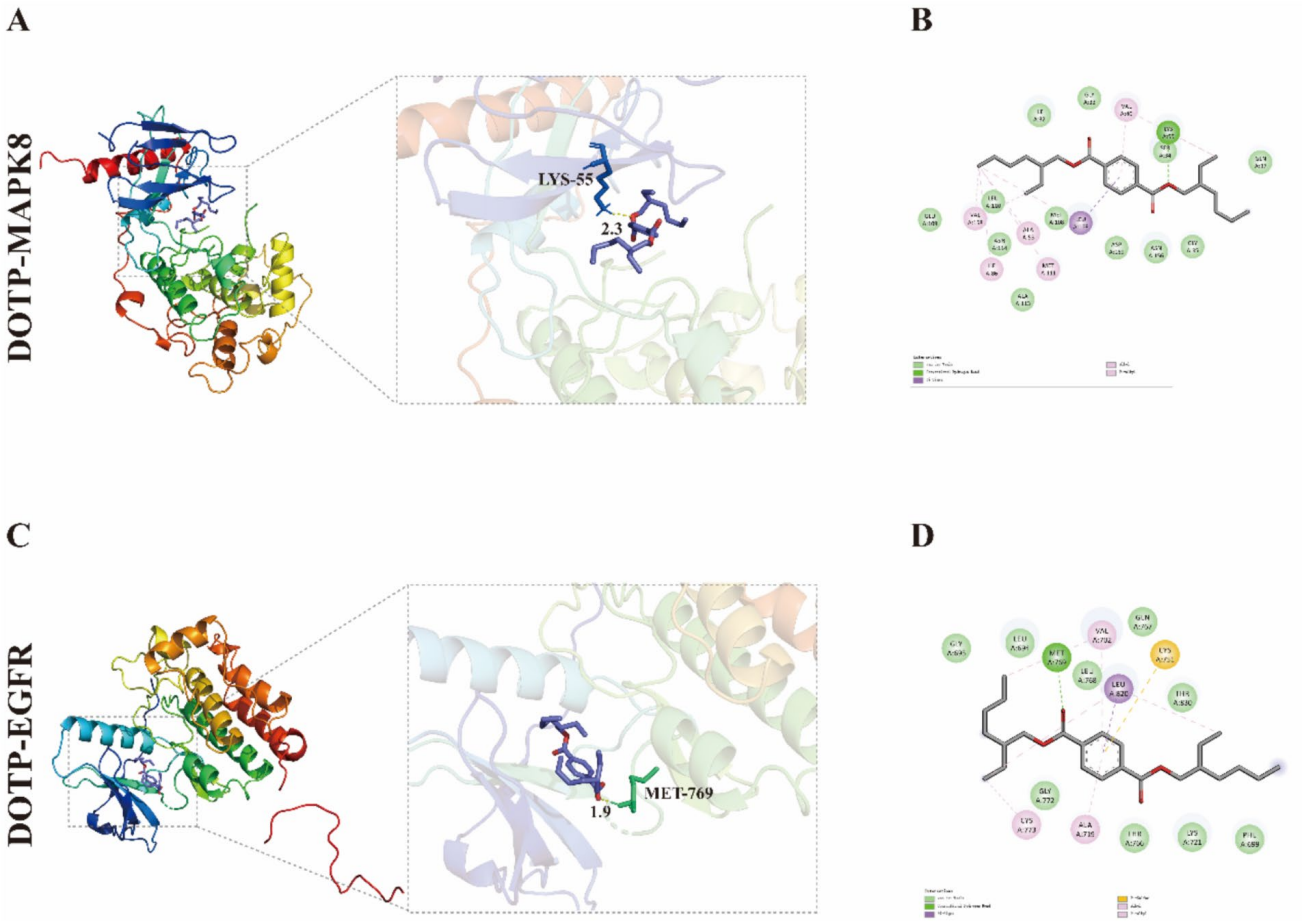
Molecular docking analysis of DOTP with MAPK8 and EGFR. **(A)** Lowest-energy docking conformation of DOTP–MAPK8 (−7.2 kcal/mol); **(B)** Two-dimensional interaction diagram of DOTP–MAPK8; **(C)** Lowest-energy docking conformation of DOTP–EGFR (−5.9 kcal/mol); **(D)** Two-dimensional interaction diagram of DOTP–EGFR.

We conducted molecular docking to investigate the potential interactions between DOTP and the seven core target proteins. Prior to evaluating DOTP, re-docking the native crystallographic ligands yielded RMSD values < 2.0 Å, confirming the structural reliability of our docking parameters. All AutoDock simulations for DOTP produced binding energies ranging from −5.2 to −7.2 kcal/mol, indicating favorable and spontaneous interactions. Importantly, these values were highly consistent with the orthogonal cross-validation results obtained via AutoDock Vina (Table 1). The lowest-energy conformations were visualized using PyMOL and Discovery Studio to elucidate the structural basis of these affinities. The 3D binding pose of the DOTP–MAPK8 complex shows the ligand occupying the active site pocket (Figure 4A; −7.2 kcal/mol). Its corresponding 2D interaction diagram (Figure 4B) details the precise intermolecular forces, revealing extensive van der Waals forces, hydrophobic interactions, and specific hydrogen bonds with critical amino acid residues (e.g., LYS-55) that stabilize the complex. Similarly, the 3D conformation of DOTP–EGFR (Figure 4C; −5.9 kcal/mol) and its 2D plot (Figure 4D) highlight key interacting residues, such as MET-769, suggesting how DOTP might allosterically or competitively interfere with EGFR kinase activity. Comparable stable interactions were visualized for DOTP–BCL2 (Supplementary Figures 1A,B; −5.5 kcal/mol), DOTP–MTOR (Supplementary Figures 1C,D; −5.5 kcal/mol), DOTP–CASP3 (Supplementary Figures 1E,F; −5.4 kcal/mol), DOTP–NFKB1 (Supplementary Figures 1G,H; −5.2 kcal/mol), and DOTP–TLR4 (Supplementary Figure 1I,J; −5.2 kcal/mol). Together, these residue-level analyses provide robust structural evidence that DOTP can directly anchor to and potentially modulate the activity of these core neurotoxicity mediators.

**Table 1:** Binding energies of ligands and receptors.

Ligand	Receptor	AutoDock4 energy [kcal/mol]	AutoDock vina energy [kcal/mol]
DOTP	MAPK8	−7.2	−7.2
DOTP	EGFR	−5.9	−5.6
DOTP	BCL2	−5.5	−5.4
DOTP	MTOR	−5.5	−5.4
DOTP	CASP3	−5.4	−5.4
DOTP	NFKB1	−5.2	−5.2
DOTP	TLR4	−5.2	−5.0

DOTP, Dioctyl terephthalate; MAPK8, Mitogen-Activated Protein Kinase 8; EGFR, Epidermal Growth Factor Receptor; BCL2, B-cell lymphoma 2; MTOR, Mechanistic Target Of Rapamycin Kinase; CASP3, Caspase 3; NFKB1, Nuclear Factor Kappa B Subunit 1; TLR4, Toll Like Receptor 4.

### Molecular dynamics simulations of the core targets

To further evaluate the dynamic stability and binding interactions between DOTP and its seven predicted neurotoxicity-associated targets (EGFR, BCL2, CASP3, MAPK8, TLR4, NFKB1, and MTOR), we conducted 100-ns all-atom MD simulations for each complex. Four commonly used metrics were employed to assess structural stability and conformational dynamics: root-mean-square deviation (RMSD), root-mean-square fluctuation (RMSF), radius of gyration (Rg), and solvent-accessible surface area (SASA).

**Figure 5:**
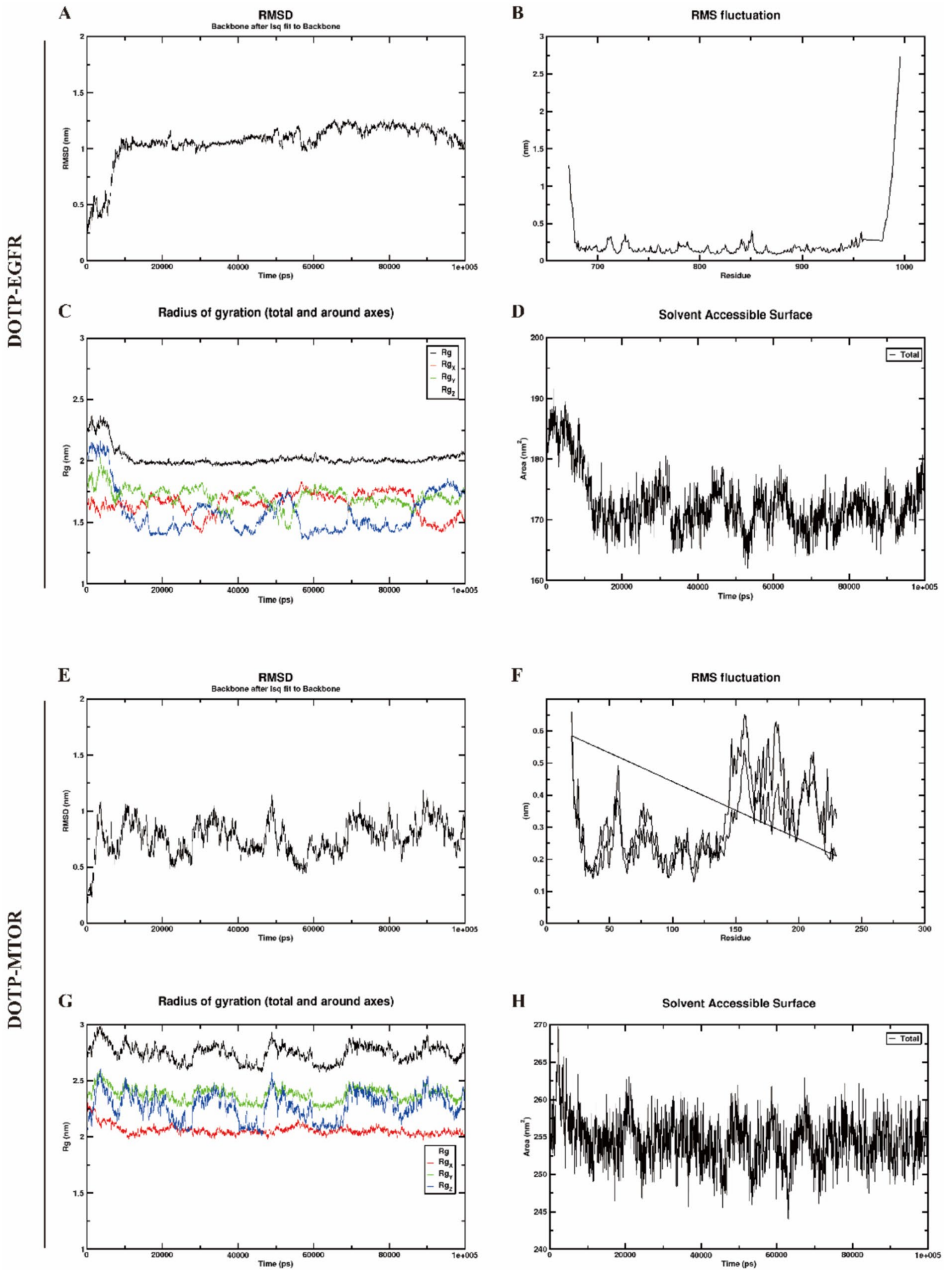
Molecular dynamics simulation results for DOTP complexes with EGFR and MTOR. **(A–D)** RMSD **(A)**, RMSF **(B)**, Rg **(C)**, and SASA **(D)** profiles for the DOTP–EGFR complex; **(E–H)** RMSD **(E)**, RMSF **(F)**, Rg **(G)**, and SASA **(H)** profiles for the DOTP–MTOR complex.

RMSD assesses the overall stability of protein–ligand complexes by tracking deviations in atomic positions over time. In our simulations, all complexes reached equilibrium during the latter stages, indicating steady binding events. The EGFR–DOTP complex stabilized within a narrow fluctuation range after approximately 50 ns, demonstrating the highest stability among the tested proteins (Figure 5A). In contrast, the BCL2–DOTP complex exhibited the greatest fluctuations (Supplementary Figure 2E), while the MTOR–DOTP complex required the longest time to equilibrate (Figure 5E). The remaining complexes demonstrated structural stabilization over the 100-ns trajectory, maintaining steady RMSD plateaus indicative of robust interactions. This included DOTP bound to MAPK8 (Supplementary Figure 2A), CASP3 (Supplementary Figure 2I), NFKB1 (Supplementary Figure 2M), and TLR4 (Supplementary Figure 2Q).

We evaluated the RMSF values of amino acid residues to identify local motions and backbone flexibility. Although overall flexibility remained low across all complexes, target-specific differences were apparent. The EGFR–DOTP complex exhibited the smallest local fluctuations (Figure 5B), reflecting a rigid backbone upon ligand binding. Conversely, the MTOR–DOTP complex showed higher RMSF values (Figure 5F), particularly within several loop regions, indicating increased local flexibility. Consistent, moderate residue fluctuations were observed for the other complexes—including MAPK8 (Supplementary Figure 2B), BCL2 (Supplementary Figure 2F), CASP3 (Supplementary Figure 2J), NFKB1 (Supplementary Figure 2N), and TLR4 (Supplementary Figure 2R)—suggesting that DOTP binding preserves core protein stability while permitting functional loop movements.

The Rg, a measure of structural compactness, was monitored throughout the simulations to evaluate global conformational changes. Each complex exhibited Rg fluctuations within a stable range. The EGFR–DOTP complex maintained the lowest and most uniform Rg profile (Figure 5C), signifying a highly compact overall structure. The MTOR–DOTP complex displayed the highest and most variable Rg values (Figure 5G), consistent with a more expanded conformation. The Rg trajectories for MAPK8 (Supplementary Figure 2C), BCL2 (Supplementary Figure 2G), CASP3 (Supplementary Figure 2K), NFKB1 (Supplementary Figure 2O), and TLR4 (Supplementary Figure 2S) remained largely steady without extreme unfolding events, indicating stable structural compactness during the interactions.

We analyzed SASA to assess changes in solvent exposure upon complex formation, which correlates directly with the extent of protein–ligand surface interaction. The EGFR–DOTP (Figure 5D) and MAPK8–DOTP (Supplementary Figure 2D) complexes exhibited the lowest and most stable SASA values, suggesting a thoroughly buried and stable hydrophobic interface. Conversely, the MTOR–DOTP complex showed the highest and most fluctuating SASA values (Figure 5H), implying that substantial surface regions remained exposed or fluctuated dynamically after binding. SASA values for BCL2 (Supplementary Figure 2H), CASP3 (Supplementary Figure 2L), NFKB1 (Supplementary Figure 2P), and TLR4 (Supplementary Figure 2T) plateaued rapidly and maintained narrow amplitude variations, confirming stable solvent interactions over time.

Overall, these MD simulation results support the hypothesis that DOTP forms stable interactions with multiple neurotoxicity-related targets. The combination of stable RMSD, moderate residue flexibility, consistent Rg, and stable SASA profiles indicates that the EGFR–DOTP complex is the most stable, compact, and least flexible, whereas the MTOR–DOTP complex is the least compact and has the greatest surface exposure. The remaining complexes exhibited intermediate stability. These computational findings provide crucial dynamic evidence that complements our molecular docking results, reinforcing the potential for DOTP to exert neurotoxic effects through multi-target interactions prior to *in vivo* experimental validation.

### DOTP exposure induces neuronal damage, neurological dysfunction, and pathway dysregulation *in vivo*

**Figure 6:**
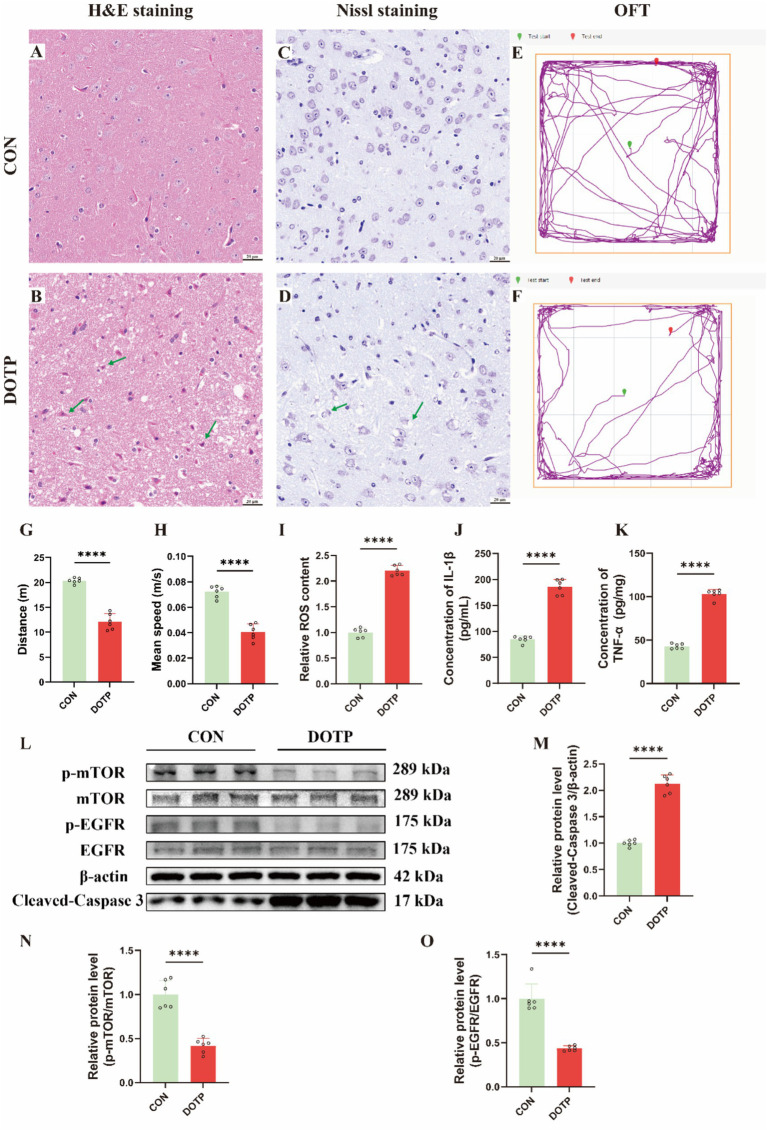
*In vivo* validation of DOTP-induced neurotoxicity, behavioral deficits, and molecular mechanism dysregulation in mice. **(A,B)** Representative H&E staining of brain tissue from the CON **(A)** and DOTP-exposed **(B)** groups; green arrows indicate irregular, condensed-nucleus neurons. **(C,D)** Representative Nissl staining from the CON **(C)** and DOTP-exposed **(D)** groups; green arrows denote morphologically abnormal neurons with chromatolysis (scale bar = 20 μm). **(E,F)** Representative locomotor trajectories in the open-field test (OFT). **(G,H)** Quantification of total distance **(G)** and mean speed **(H)** in the OFT. **(I)** Relative ROS content in brain tissues. **(J,K)** Concentrations of pro-inflammatory cytokines IL-1*β* **(J)** and TNF-*α* **(K)** measured by ELISA. **(L)** Western blot analysis of p-mTOR, mTOR, p-EGFR, EGFR, Cleaved-Caspase 3, and β-actin. **(M–O)** Quantitative analysis of relative protein levels for Cleaved-Caspase 3/β-actin **(M)**, p-mTOR/mTOR **(N)**, and p-EGFR/EGFR **(O)**. Data are presented as mean ± SEM (*n* = 6 per group). ****p < 0.0001 compared to the CON group.

To investigate the neurotoxic effects of DOTP in vivo, we performed comprehensive histological, behavioral, and molecular analyses. H&E staining of the cerebral cortex revealed a normal, orderly histological appearance in control mice (Figure 6A). In contrast, DOTP-treated mice exhibited evident neuronal damage, characterized by irregular cell morphology, nuclear condensation, and disorganized tissue architecture (Figure 6B). Nissl staining corroborated these structural deficits. While control brains displayed abundant Nissl bodies (Figure 6C), DOTP-exposed animals showed a marked reduction in Nissl substance (Figure 6D), a hallmark of chromatolysis. Neurological function was evaluated using the open-field test. Representative tracking maps illustrated active exploration in controls (Figure 6E), whereas DOTP-treated mice exhibited restricted movement (Figure 6F). Quantitative analysis confirmed that DOTP exposure significantly reduced both the total distance traveled (Figure 6G) and the mean velocity (Figure 6H).

To directly validate the specific molecular pathways predicted by our in silico model, we assessed key mechanistic biomarkers. Biochemical assays confirmed the pronounced accumulation of ROS (Figure 6I) and elevated concentrations of the pro-inflammatory cytokines IL-1β and TNF-*α* (Figures 6J,K) in DOTP-treated cortices. Consistent with the in silico predictions, Western blot analysis revealed a significant upregulation of the apoptotic executioner cleaved caspase-3 (Figures 6L,M). Furthermore, while the total expression levels of EGFR and mTOR remained stable, the phosphorylation ratios of p-mTOR/mTOR (Figure 6N) and p-EGFR/EGFR (Figure 6O) were significantly suppressed in the DOTP group. These *in vivo* molecular data provide direct evidence validating the three predicted mechanistic axes: oxidative stress, neuroinflammation, and synaptic/neurotrophic signaling dysregulation.

## Discussion

In this study, we systematically identified 90 potential targets associated with DOTP-induced neurotoxicity by integrating compound- and disease-related data from extensive databases. Through PPI network construction and topological analysis, we extracted seven key nodes—EGFR, BCL2, CASP3, MAPK8, TLR4, NFKB1, and MTOR—as central mediators. These molecules were subsequently selected for molecular docking and MD simulations based on their network centrality and documented relevance to neuroinflammatory and apoptotic signaling (Glass et al., 2010; Czabotar et al., 2014; Galluzzi et al., 2018). Crucially, we transitioned from in silico predictions to in vivo biological validation by conducting comprehensive behavioral, histological, and targeted molecular assays (including Western blotting and ELISA). This integrative strategy provides deep mechanistic insights into the neurotoxic potential of DOTP and aligns with recent paradigm shifts that emphasize combining network toxicology with structural predictive simulations to accurately elucidate the neurological risks of emerging environmental chemicals (Hopkins, 2008).

Although DOTP has been widely introduced as a structurally favorable, “safe” alternative to traditional legacy phthalates like DEHP (Bui et al., 2016), our integrative model treats it as a potentially active neurotoxicant rather than a biologically inert substitute. This framing is motivated by biomonitoring evidence indicating increasing population exposure to DOTP across multiple regions following the phase-down of regulated ortho-phthalates (Lessmann et al., 2016). Consistent with the mechanistic themes underlying legacy phthalate neurotoxicity (Engel et al., 2010; Grandjean and Landrigan, 2014), our combined computational and in vivo analyses support a multi-pathway hypothesis wherein DOTP converges on three interconnected axes: oxidative stress-linked apoptosis, receptor-proximal neuroinflammation, and synaptic/neuronal maintenance dysregulation.

First, the targeted disruption of MAPK8, BCL2, and CASP3 points toward an oxidative stress-associated apoptotic signaling cascade. As extensively documented in neurotoxicology, the accumulation of reactive oxygen species (ROS) is a primary initiating event that overwhelms anti-apoptotic buffering (e.g., BCL2) and triggers executioner caspases, leading to irreversible neuronal demise (Dhanasekaran and Reddy, 2008; Tait and Green, 2010). Our in vivo molecular data profoundly mirror this trajectory. Rather than merely validating computational affinities, the massive surge in ROS and the highly significant upregulation of active cleaved-Caspase-3 observed in DOTP-exposed murine cortices provide a definitive biochemical basis for the severe neuronal pyknosis and cytoarchitectural disruption seen in our histological evaluations. This firmly establishes a causal molecular link between DOTP exposure and oxidative neurodegeneration.

Second, the stable engagement of DOTP with TLR4 and NFKB1 highlights a pronounced neuroinflammatory component. The TLR4/NF-κB axis is a well-characterized route for microglial inflammatory activation and subsequent cytokine storms, frequently emphasized as a core mechanism in environmental neurotoxicity (Kawai and Akira, 2010; Hayden and Ghosh, 2012). The marked increase in pro-inflammatory cytokines IL-1β and TNF-*α* within the cortices of DOTP-treated mice substantiates our in silico hypothesis that DOTP may directly provoke receptor-proximal inflammatory responses. This robust cytokine signaling provides a compelling mechanistic explanation for the bystander neuronal injury and neuroinflammation frequently observed during chronic plasticizer exposure (Block et al., 2007).

Third, the dysregulation of EGFR and MTOR reveals a critical breakdown in neuronal maintenance and synaptic plasticity. EGFR and mTOR are master regulators governing neurotrophic signaling, protein synthesis, and autophagic clearance in the central nervous system (Laplante and Sabatini, 2012; Saxton and Sabatini, 2017). Dysregulation of these kinases represents a convergent vulnerability in cognitive decline and synaptic impairment (Hoeffer and Klann, 2010). Importantly, our Western blot analyses revealed that while total EGFR and mTOR protein levels remained stable, their active phosphorylated forms (p-EGFR and p-mTOR) were dramatically suppressed following DOTP exposure. This functional blockade of mTOR-dependent protein synthesis offers a rigorous molecular explanation for the chromatolysis—manifested as the marked reduction of Nissl bodies (the rough endoplasmic reticulum)—and the subsequent behavioral locomotor deficits observed in our murine models (Hoeffer and Klann, 2010). Collectively, these highly integrated structural, behavioral, and molecular findings argue for a neuro-focused evaluation of DOTP, rather than relying on structural substitution as a proxy for safety.

From a methodological perspective, evaluating environmental plasticizers computationally presents unique conceptual challenges. DOTP is fundamentally a hydrophobic molecule lacking the distinct polar pharmacophoric motifs typical of endogenous physiological ligands or highly targeted drugs. Consequently, the docking affinities observed in our study (−5.2 to −7.2 kcal/mol) should not be strictly interpreted as highly specific lock-and-key pharmacological interactions. Rather, DOTP likely functions as a lipophilic disruptor, stably occupying hydrophobic binding crevices primarily via van der Waals forces, thereby non-specifically altering physiological conformations. Furthermore, while our multi-tiered approach effectively bridges computational predictions with functional *in vivo* validation, certain limitations warrant consideration. Future comprehensive evaluations should incorporate unbiased stereological morphometry, multi-omics sequencing (e.g., transcriptomics), and robust dose–response longitudinal designs to fully delineate the dynamic toxicokinetics of DOTP. Additionally, expanding computational methodologies to include multiple independent MD trajectories and free energy calculations (Hollingsworth and Dror, 2018) will further refine these predictive structural models.

## Conclusion

In summary, the integration of network toxicology, molecular docking, and MD simulations enabled a comprehensive exploration of the mechanisms underlying DOTP-induced neurotoxicity. The predicted neurotoxic phenotype was further corroborated by in vivo histological, molecular, and behavioral evidence. This multiscale approach not only enhances mechanistic hypothesis generation for chemical toxicity screening but also underscores the practical utility of combining computational predictions with experimental validation. Future research should integrate large-scale omics data, longitudinal exposure models, and advanced behavioral paradigms to further delineate the neurotoxic risks posed by emerging environmental pollutants such as DOTP.

## Data Availability

The original contributions presented in the study are included in the article/Supplementary material, further inquiries can be directed to the corresponding authors.

## Footnotes

1
https://pubchem.ncbi.nlm.nih.gov/

2
https://sea.bkslab.org/

3
https://prediction.charite.de/

4
http://swisstargetprediction.ch/

5
https://www.genecards.org/

6
https://db.idrblab.net/ttd/

7
https://www.omim.org/

8
https://www.rcsb.org/
