# Supplementary material for: Effective analysis of neurotoxicity and mechanisms of dioctyl terephthalate using network toxicology
Source: Front Neurosci. 2026 Apr 10;20:1783807. doi: 10.3389/fnins.2026.1783807 (PMC13106157; doi:10.3389/fnins.2026.1783807)
Supplement: Supplementary file 1 [file Data_Sheet_1.docx]

**Effective analysis of neurotoxicity and mechanisms of Dioctyl terephthalate using network toxicology**

Hao Tang^a,1^, Ting Qin^b,1^, Qinghuan Yang^c,1^, Zhiwen Yan^c^, Changqing Li^a^, Wuyang He^d*^, Yinghong Tang^e*^

^a^ Department of Neurology, The Second Affiliated Hospital of Chongqing Medical University, Chongqing, China

^b^ Department of Respiratory Disease, Science City People's Hospital, Chongqing, China

^c^ Department of Neurology, The First Affiliated Hospital of Chongqing Medical University, Chongqing, China

^d^ Department of Oncology, The Second Affiliated Hospital of Chongqing Medical University, Chongqing, China

^e^ Department of Gerontology, The Second Affiliated Hospital of Chongqing Medical University, Chongqing, China

**^1^ these authors contributed equally to this work**

**^*^ Corresponding Author:**

Wuyang He^d*^

Linjiang Road, Yuzhong District, Chongqing, China

Email address: [hewuyang@hospital.cqmu.edu.cn](mailto:hewuyang@hospital.cqmu.edu.cn)

Yinghong Tang^e*^

Linjiang Road, Yuzhong District, Chongqing, China

Email address: [tangyh@hospital.cqmu.edu.cn](mailto:tangyh@hospital.cqmu.edu.cn)


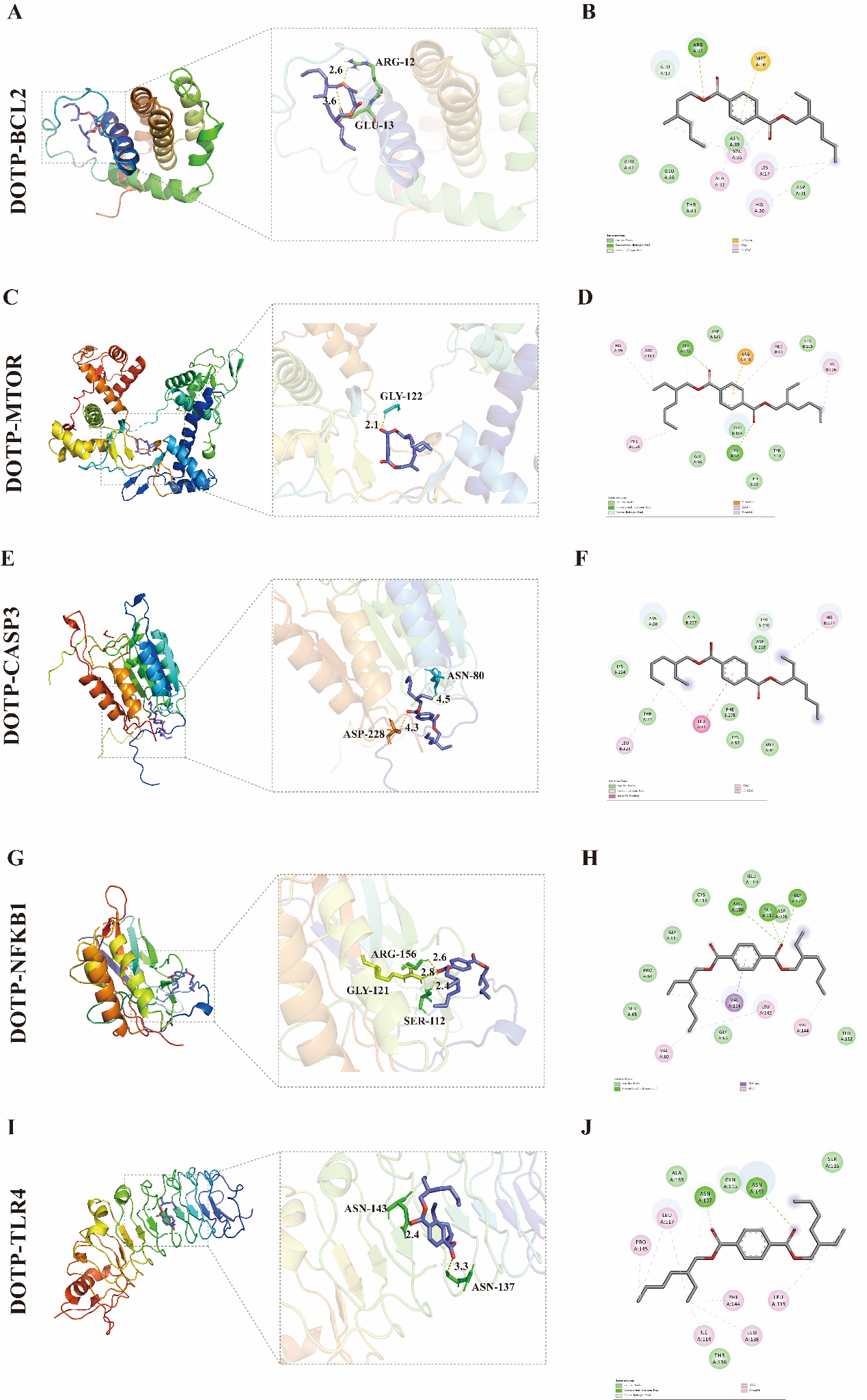


Supplementary Fig. 1. Molecular docking of DOTP with BCL2, MTOR, CASP3, NFKB1, and TLR4. (A) Lowest-energy docking conformation of DOTP–BCL2 (–5.5 kcal/mol); (B) Two-dimensional interaction diagram of DOTP–BCL2; (C) Lowest-energy docking conformation of DOTP–MTOR (–5.5 kcal/mol); (D) Two-dimensional interaction diagram of DOTP–MTOR; (E) Lowest-energy docking conformation of DOTP–CASP3 (–5.4 kcal/mol); (F) Two-dimensional interaction diagram of DOTP–CASP3; (G) Lowest-energy docking conformation of DOTP–NFKB1 (–5.2 kcal/mol); (H) Two-dimensional interaction diagram of DOTP–NFKB1; (I) Lowest-energy docking conformation of DOTP–TLR4 (–5.2 kcal/mol); (J) Two-dimensional interaction diagram of DOTP–TLR4.


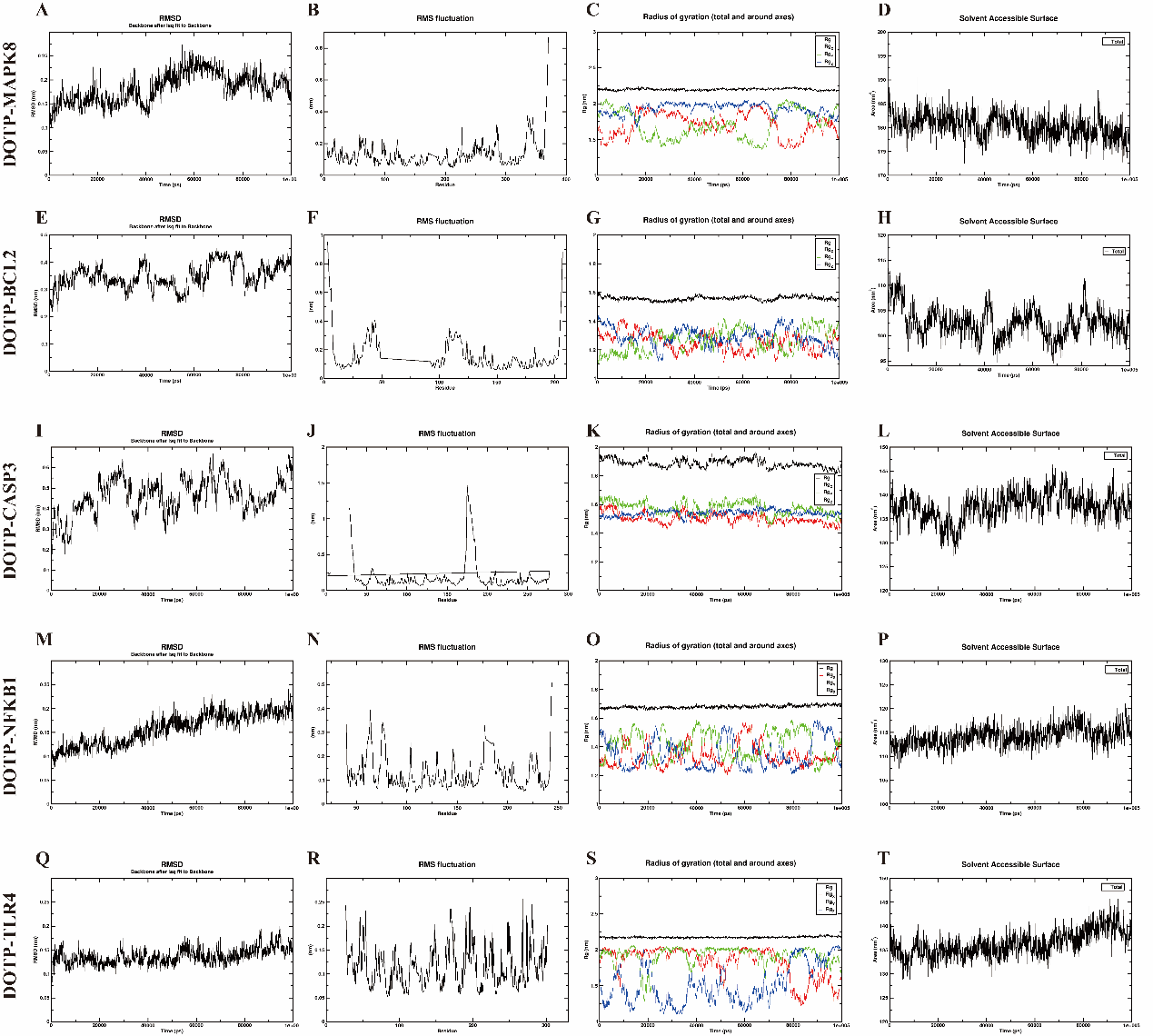


Supplementary Fig. 2. Molecular dynamics simulation results for DOTP complexes with MAPK8, BCL2, CASP3, NFKB1, and TLR4. (A-D) RMSD (A), RMSF (B), Rg (C), and SASA (D) profiles for the DOTP–MAPK8 complex; (E-H) RMSD (E), RMSF (F), Rg (G), and SASA (H) profiles for the DOTP–BCL2 complex; (I-L) RMSD (I), RMSF (J), Rg (K), and SASA (L) profiles for the DOTP–CASP3 complex; (M-P) RMSD (M), RMSF (N), Rg (O), and SASA (P) profiles for the DOTP–NFKB1 complex; (Q-T) RMSD (Q), RMSF (R), Rg (S), and SASA (T) profiles for the DOTP–TLR4 complex;
